# Discontinuous EBOV RNA synthesis events in patients with Ebola virus disease and their relationship to viral load and outcome of infection

**DOI:** 10.1128/jvi.00826-25

**Published:** 2025-11-11

**Authors:** Xiaofeng Dong, Isabel Garcia-Dorival, Natasha Y. Rickett, Andrew Bosworth, Sophie Smither, Stuart Dowall, Miles W. Carroll, David A. Matthews, Thomas R. Laws, Paul Digard, Julian A. Hiscox

**Affiliations:** 1 Institute of Infection, Veterinary and Ecological Sciences, University of Liverpool 4591, Liverpool, United Kingdom; 2 National Center of Microbiology (CNM), Instituto de Salud Carlos III (ISCIII) 38176, Madrid, Spain; 3 Regional Public Health Laboratory, UK Health Security Agency, University Hospitals, Birmingham NHS Foundation Trust 1730, Birmingham, United Kingdom; 4 Defence Science and Technology Laboratory 13330, Porton Down, United Kingdom; 5 UK Health Security Agency 371011, Porton Down, United Kingdom; 6 Pandemic Sciences Institute & Centre for Human Genetics, University of Oxford 6396, Oxford, United Kingdom; 7 School of Cellular and Molecular Medicine, University of Bristol 1980, Bristol, United Kingdom; 8 Roslin Institute, University of Edinburgh 3124, Edinburgh, United Kingdom; 9 A*STAR Infectious Diseases Labs (ID Labs) 670853 , Singapore, Singapore; The Ohio State University, Columbus, Ohio, USA

**Keywords:** defective genomes, Ebola, viral replication, transcriptomics, Ebola virus

## Abstract

Ebola virus (EBOV) is a negative strand RNA virus that causes Ebola virus disease (EVD) with a high case fatality rate. During the acute phase of infection, a subsequent fatal outcome is characterized by an increased upregulation of interferon and inflammatory pathways compared to survivors. Replication of genomes from negative strand RNA viruses (and RNA viruses in general) can result in the generation of defective genomes that may interfere with viral replication and stimulation of the innate immune response. We characterized the presence of defective genomes in blood samples from humans who were positive for EBOV and processed by the European Mobile Laboratory during the 2013–2016 West African EVD outbreak. A bioinformatics tool, DI-tector, was used to identify sequence motifs associated with the four different types of defective genomes. The analysis indicated that sequence features indicative of defective genomes were present in blood samples in patients during the acute phase of infection. The most common type of defective genome identified was insertion followed by deletion, 5′ copy back and then 3′ copy back. In general, the abundance of defective genomes correlated with viral load, but particularly with patient outcome. We postulate that the presence of defective genomes correlates with an upregulation in the interferon response and resultant inflammation and may, therefore, be an important contributory factor in patients with severe EVD.

**IMPORTANCE:**

EBOV and filoviruses in general are high consequence infectious diseases whose outbreaks can severely impact the lives of those affected. In this study, we show that during EBOV replication in humans, defective genomes can be produced, which complements previous studies in nonhuman primate models of disease and in cell culture. The abundance of these defective genomes correlates with disease outcome in acutely ill patients. In people who go on to die from EVD, they appear to have higher levels of defective genomes than in people who go on to survive infection. This may, in turn, cause a greater upregulation of interferon and inflammation, which are some of the biggest factors in determining disease severity and adverse patient outcome. Therefore, we caution the potential use of defective genomes as a therapy for EVD, as has been proposed for other negative strand RNA viruses.

## INTRODUCTION

Negative strand RNA viruses contain examples of some of the most troubling human pathogens. These viruses can be divided into two groups depending on whether they have segmented or non-segmented genomes. Those with non-segmented genomes form the *Mononegavirales* and include EBOV, respiratory syncytial virus, measles virus, and rabies virus. In general, the genome architecture for these viruses is similar, with replication signals located at the 3′ and 5′ ends, followed from the 3′ end with a transcriptional start site, coding sequence and transcriptional stop site (1). Genes are arranged in a linear manner along the genome and (mostly) sequentially transcribed from the 3′ end to the 5′ end.

During the replication of RNA virus genomes, discontinuous RNA synthesis can occur. This generates new viral RNA species with internal deletions or insertions, resulting in defective genomes. In the *Mononegavirales,* examples of defective genomes have been defined for EBOV (2), measles virus (3), and parainfluenza virus 5 (4). EBOV defective genomes have been identified in non-human primate models of disease (5) and in a human patient and survivor of EVD (6). Defective genomes are composed of shorter lengths of RNA that contain intact 3′ and 5′ ends (and, thus, replication signals) but contain deletions of coding and/or non-coding sequence. These molecules can then be replicated alongside the full-length wild-type (WT) genome during virus infection. When defective genomes compete with the WT genome, they are referred to as defective interfering genomes (DIs). Defective genomes can compete with the authentic viral RNA segments for the RNA-dependent RNA polymerase (RdRp) and other viral proteins, hence resulting in a reduction of virus infectivity through the competition for limited resources (7). They can also be incorporated into virus particles reducing the particle to infectivity ratio (8). Defective genomes have generally been identified through serial passage of viruses at high multiplicity of infections in cell culture systems and may influence infection. They have also been proposed to facilitate persistent infection in patients who have had measles virus infection (9) or EVD (2, 3, 5, 10, 11). Although in one EBOV survivor, defective genomes could be identified, data suggested that a recrudescent infection was potentially triggered by minimal viral replication, rather than through competition between intact and defective genomes (6). Defective genomes may also interact with, and stimulate, the host response to virus infection. In a number of viruses, including parainfluenza virus 5, defective genomes have been shown to activate the interferon cascade (4).

There are four main types of defective genomes: deletion, insertion, 5′ and 3′ copyback. Defective genomes formed through deletion are shortened forms of the parental virus genome that share the 3′ and 5′ ends with the original virus genome sequence. A defective genome with an insertion(s) can result if the re-initiation site occurs at a position that is 3′ to the break point. Copy-back DIs, and the associated snap-back DIs, comprise a piece of the viral genome between reverse complementary versions of its 5′ end. Copy-back DIs occur when the viral polymerase detaches from the template and reattaches to the newly synthesized strand, copying back the 5′ end of the genome. These lead to different features that can be identified by sequencing (12) and have been used in the identification of defective genomes in animal models of EVD (5) or in a human patient (6).

Given that defective genomes may interfere with virus replication and/or stimulate the host response, then the presence of these RNA molecules in patients with EVD may influence outcome, i.e., either death or survival. However, EVD may be influenced by several factors including the host response (13, 14) and the presence of other infections at the time of acute symptoms (15). The single biggest contributory factor appears to be viral load (16, 17). With higher viral loads being correlated with a fatal outcome. During the acute phase of EVD, West African patients who had a fatal infection showed a stronger upregulation of interferon signaling compared to survivors (13). Whether this was caused by fundamental genotypic differences between different patients or potentially related to virus genetics (or a mixture of both) is unknown. Defective genomes have been shown to protect against influenza A virus infection *in vivo* (18–20), potentially through competition that reduces viral load (20). Therefore, one hypothesis is that in acute EVD patients with lower viral loads who went on to survive, they may have had significant populations of defective EBOV genomes. Conversely, given the relationship between the presence of defective genomes and the interferon response (4), an alternative hypothesis would be that fatal EBOV infections had a greater number of defective EBOV genomes compared to acute survivors and, thus, elevated levels of inflammation.

To investigate whether defective genomes could be identified in humans infected with EBOV and to test between these hypotheses, RNA sequencing data from patients with EVD were interrogated using the DI-tector algorithm (12). Using the default setting, the DI-tector reports the defective genomes deletion derived from more than one nucleotide insertion/deletion in the sequencing data. Analysis of sequence reads from samples taken from patients infected with EBOV identified reads mapping to the viral genome that were indicative of defective RNA species. These species had the potential to be transmitted vertically between a mother and baby. The presence of defective genomes correlated with a high viral load and fatal outcome in patients, suggesting that the defective genomes may be a contributory factor in the higher levels of interferon signaling observed in acute patients who went on to die (13).

## RESULTS

Identifying defective RNAs in samples from patients in outbreak settings can be challenging. Generally, the RNA is of poorer quality than cell culture or animal model-derived material and, therefore, precludes a more conventional analysis using long read direct RNA sequencing, RT-PCR analysis, northern blot, or metabolic labeling. In order to investigate the presence of potential defective genomes in patients infected with EBOV, a software algorithm called DI-tector (12) was used to interrogate next-generation (RNAseq) data obtained by transcriptomic analysis of blood samples from patients with EVD. DI-tector matched segmented reads on a reference EBOV genome to infer the four different types of defective genomes. The raw sequencing data was taken from a previous analysis of viral genome evolution during the West African outbreak that we conducted (15). This general approach had been previously used to identify different species of defective genomes from sequencing data of samples from non-human primate models of EBOV, Marburg virus (MARV) and Sudan virus (SUDV) (5) and a human patient with a recrudescent infection (21).

### Defective genomes were identified in blood samples from patients with EVD

**Fig 1:**
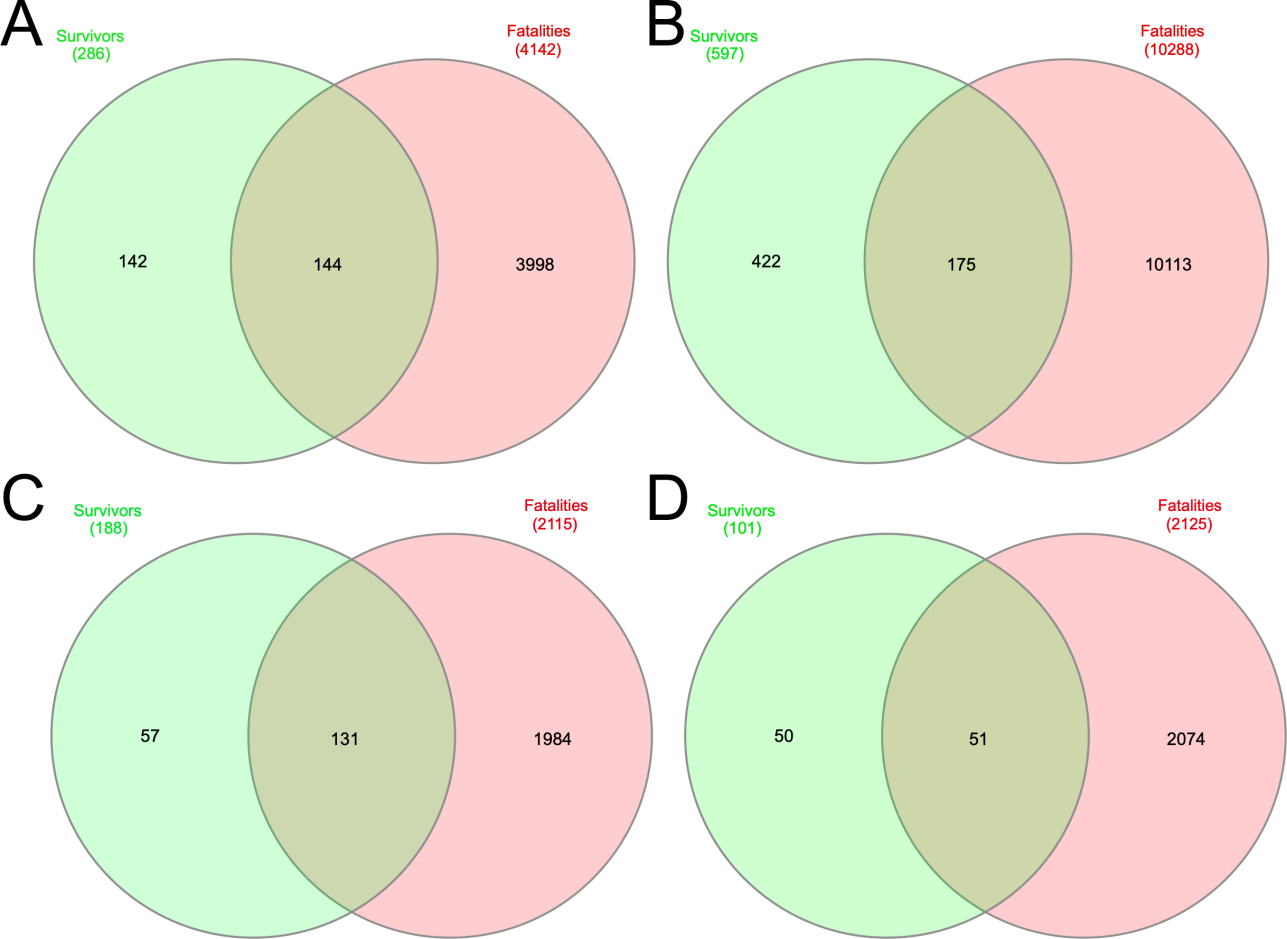
Distribution of four types of EBOV defective genomes in samples from patients with EVD. DI-tector was used to identify potential defective genomes taken from sequencing data derived from two different cohorts of patients with EVD. Blood samples were taken upon presentation and during the acute phase. Venn diagrams show the number of potential shared and unique defective genomes in EBOV patients that were either hospitalized fatal or hospitalized survivor cases. The four different types of defective genomes were (**A**) deletion, (**B**) insertion, (**C**) 5′ copy back, and (**D**) 3′ copy back.

During the West African EBOV outbreak, we used Illumina sequencing to map the evolution of the virus over the course of nearly a year (16). RNA was extracted from blood samples initially taken for diagnostic purposes, from patients upon presentation at an EBOV treatment unit. The patients had acute undiagnosed febrile illness, and the samples were tested for EBOV under the auspices of the European Mobile Laboratory (22). These patients either went on to have a fatal infection (termed hospitalized fatal) or survived (termed hospitalized survivor). Patients who were hospitalized survivors tended to have lower viral loads (Ct > 23.2) on testing than hospitalized fatal cases (Ct < 18.1) (17). Patients with viral loads around Ct = 20 had a roughly equal chance of living or dying (17). In the Illumina based sequencing approach used, generally higher viral loads correlated with a greater number of sequence reads mapping to the EBOV genome and an adverse outcome (16). To investigate whether defective genomes were present, the raw sequence reads from the blood transcriptome of 92 patients who were hospitalized fatal cases and 56 patients who were hospitalized survivors, was mined using DI-tector. This was used to identify sequence reads that were indicative of different types of potential defective genomes. Examples of the four different types of defective genomes were identified in both categories of patients, with the most common being an insertion defective genome, then followed by deletion, 5′ copy back and 3′ copy back defective genomes (Fig. 1). While some of the different defective genomes were common to both hospitalized survivor and hospitalized fatal cases, a greater number of different defective genomes were identified in hospitalized fatal cases (Fig. 1). One hypothesis is that the number of different defective genomes is related to the higher viral load observed in these patients.

**Fig 2:**
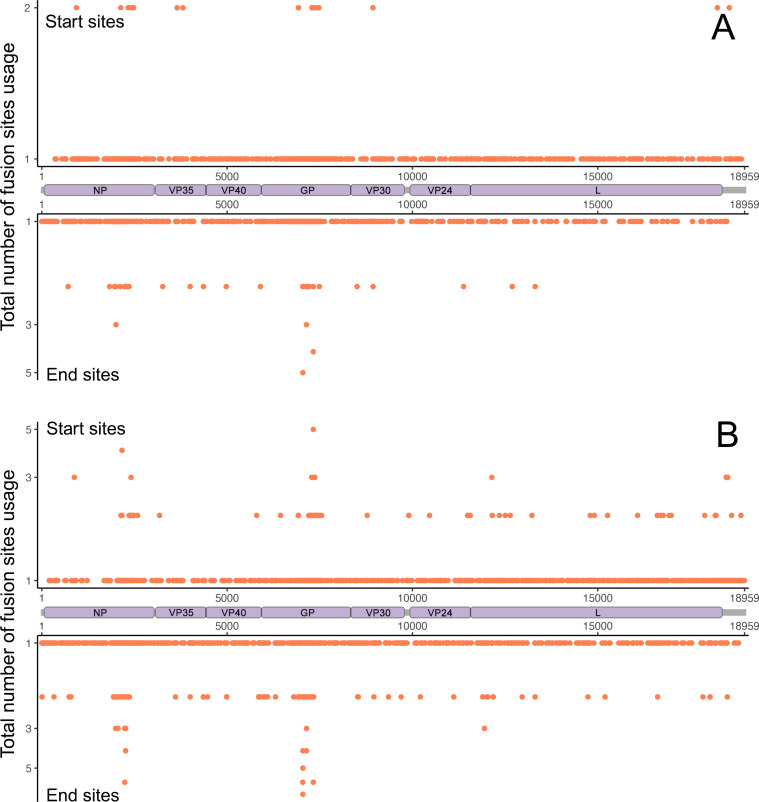
Location of the fusion sites in the insertion events along the EBOV genome. Scatter plots showing the total number of the fusion sites (start and end sites) in the insertion events along the EBOV genome identified from (**A**) hospitalized survivors and (**B**) hospitalized fatal cases.

The location of discontinuous synthesis events associated with the different defective genomes appeared to be distributed across the EBOV genome although certain fusion sites (defective start or end locations) were more frequently used than others and were specific to each type of defective genome (Fig. 2; Fig. S1 to S3). Several fusion sites clustered in the NP and GP regions were shared by multiple genomes with insertion and deletion defects, particularly in hospitalized fatal cases (Fig. 2; Fig. S1). This suggested these fusion sites were favorable in the formation of insertion and deletion defective genomes, potentially associated with selection pressure from the host. These fusion sites could also indicate some novel RNA editing sites in the viral genome.

### The presence of defective genomes correlated with a higher viral load and a fatal outcome

**Fig 3:**
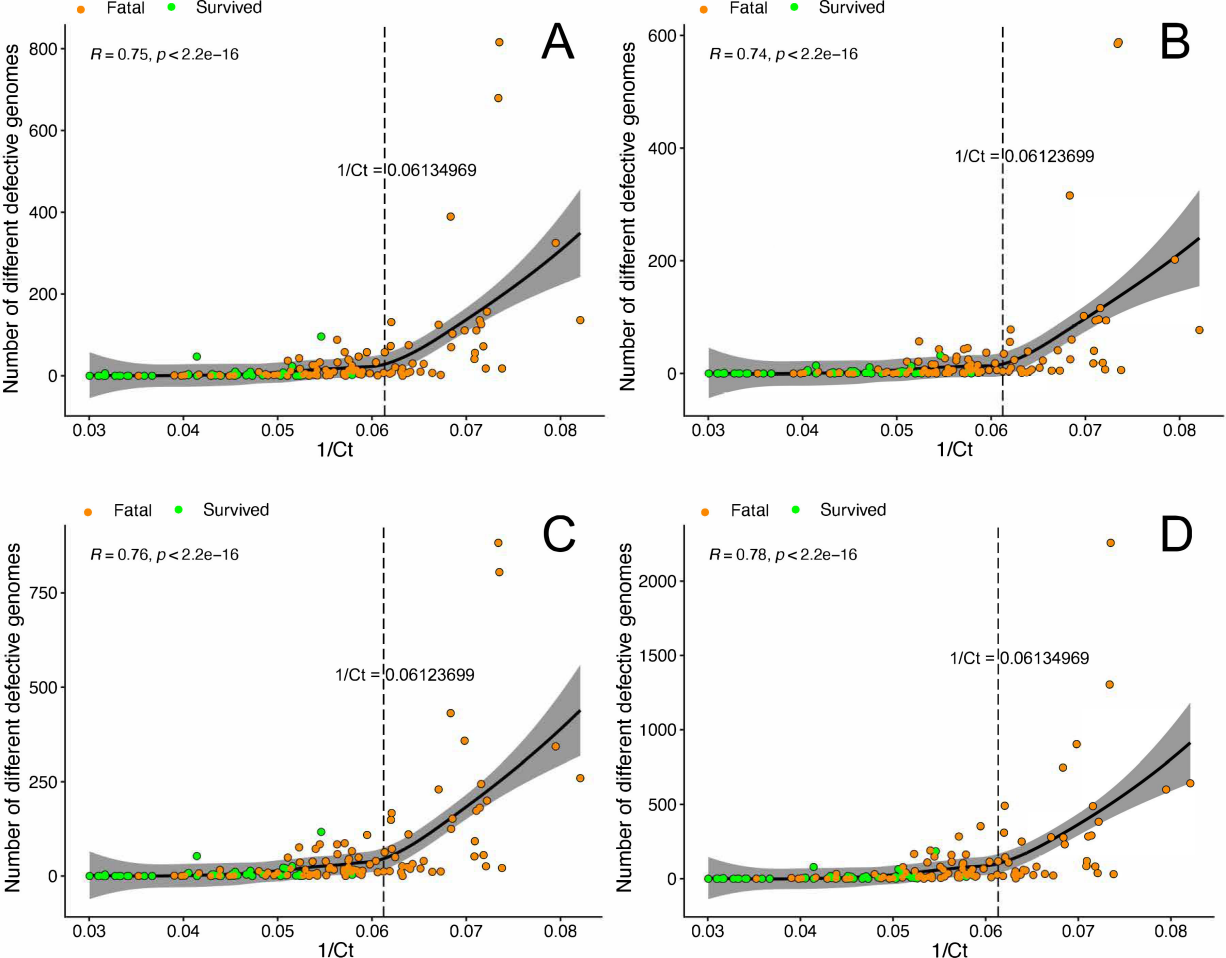
Relationship of different types of defective genomes, viral load as measured by RT-PCR and outcome. A one-sided (greater) Spearman rank correlation test was used to estimate the correlation between defective genomes (**A**: deletion, **B**: insertion, **C**: 5′ copy back, and **D**: 3′ copy back) and viral load (1/EBOV Ct) for patients between hospitalized fatal cases and hospitalized survivors, where the *R* value is the correlation coefficient ranging from −1 (strong negative correlation) to +1 (strong positive correlation), and *P* is the *P*-value for this test. The long dash line indicates the critical point. The solid black line represents the line of regression, including a 95% confidence region (light gray area).

Analysis of defective genomes in blood samples from 56 patients who were hospitalized survivors, and 92 patients who were hospitalized fatal cases, suggested that there was a positive correlation between viral load, outcome, and the number of defective genomes (Fig. 3). Although this correlation analysis indicated the higher viral load (lower Ct-value) the greater the variety of defective genomes, we found there was a critical point around 1/Ct = 0.061 (i.e., Ct = 16) in viral load for each type of defective genome where the number of different defective genomes increased exponentially (Fig. 3). The slopes of the regression lines in Fig. 3 were all close to zero, indicating that viral load was not necessarily a determinant of the abundance of defective genomes before the critical point. However, after crossing the critical point, the viral density in the infected host may enhance the formation of defective genomes. Among the 92 hospitalized fatal cases, 34 had a viral load (1/Ct) higher than the critical point, whereas no hospitalized survivors had a viral load above this threshold (Table S1). The abundance of these defective genomes may be more harmful than just EBOV alone and could contribute to progression to a fatal outcome.

**Fig 4:**
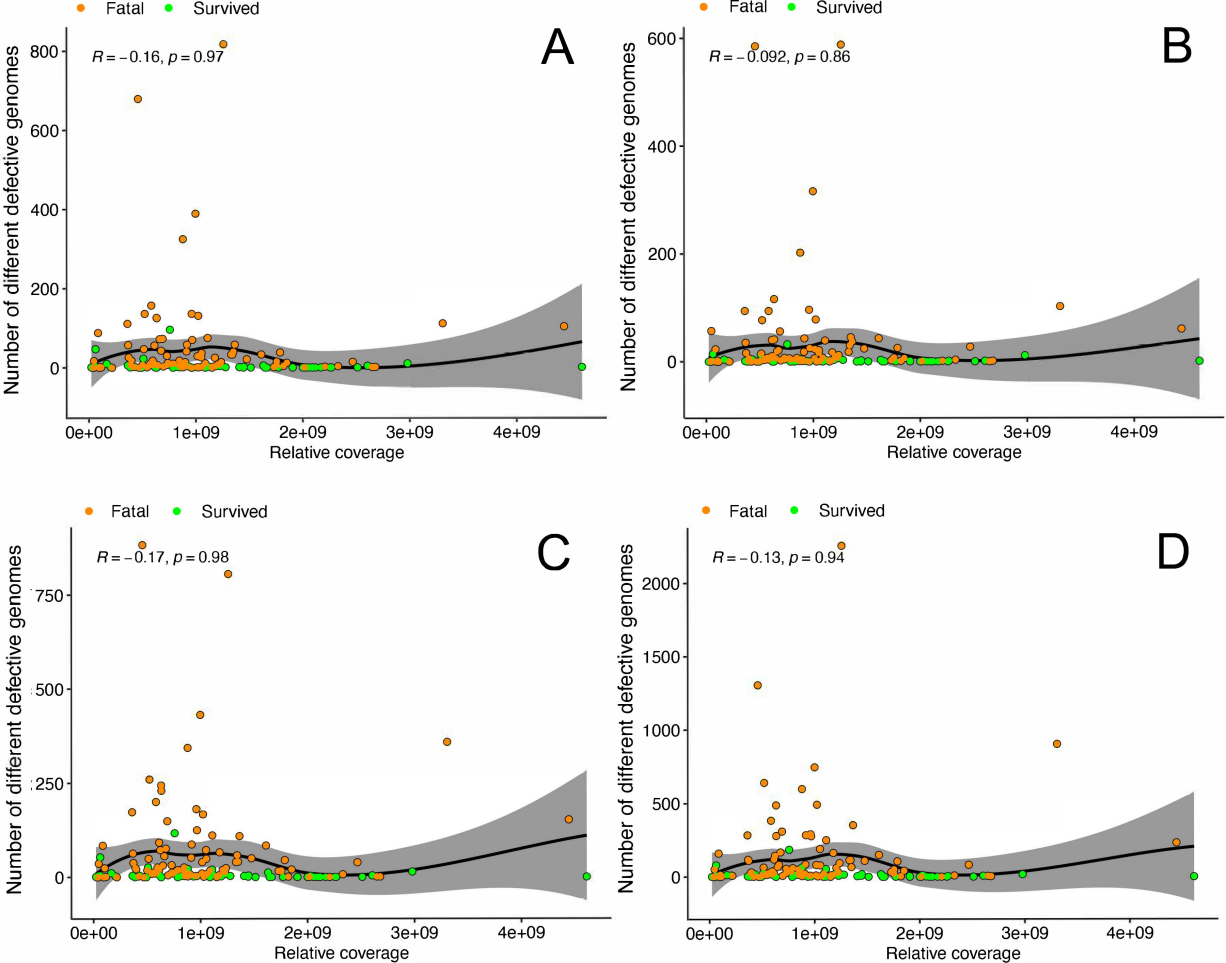
Relationship of different types of defective genomes and average sequence read depth in samples from EBOV patients. A one-sided (greater) Spearman rank correlation test was used to estimate the correlation between number of different defective genomes (**A**: deletion, **B**: insertion, **C**: 5′ copy back, and **D**: 3′ copy back) and relative sequencing converge for patients between hospitalized fatal cases and hospitalized survivors, where the *R* value is the correlation coefficient ranging from −1 (strong negative correlation) to +1 (strong positive correlation), with *P*-value stated. The solid black line represents the line of regression, including a 95% confidence region (light gray colored).

A PCR cycling/sequencing error, particularly for deletion or insertion defective genomes, may result in the identification of false positive defective genomes by DI-tector. If this was the case, then the higher the sequencing read depth the greater the number of errors – and, therefore, misidentifications. To investigate this, the relative sequencing coverage of the viral genome (the total read coverage of virus genome divided by 1/Ct) in each patient was compared to the number of different defective genomes identified in that patient by DI-tector. The relative sequencing coverage could directly reflect how many times each virus genome was sequenced in a host. The data indicated that there was no correlation between sequence read depth and the number of defective genomes detected, regardless of patient outcome (Fig. 4).

### Secondary structures of defective genomes

Structural features in viral RNA genomes, through the formation of double-stranded RNA and/or other types of folding, can act as pathogen-associated molecular patterns (PAMPs) and be detected by host pattern recognition receptors (PRRs), triggering the innate immune response (23). Single-stranded RNA genomes or transcripts from viruses may fold back on themselves, forming internal base-pairing, thus creating secondary RNA structures (23). Defective genomes can fold into specific secondary structures that may differ from the original EBOV genome, and formation of these structures may influence the host response – perhaps through activation of PRRs. Base pairing probabilities in both the original EBOV genome and these potential defective genomes (deletion, 5′ copy-back, and 3′ copy-back) were analyzed using the ScanFold approach (24, 25). (Again, we note that Di-tector infers a defective genome based on a particular sequence motif, and the sequence of the complete defective was not identified)

**Fig 5:**
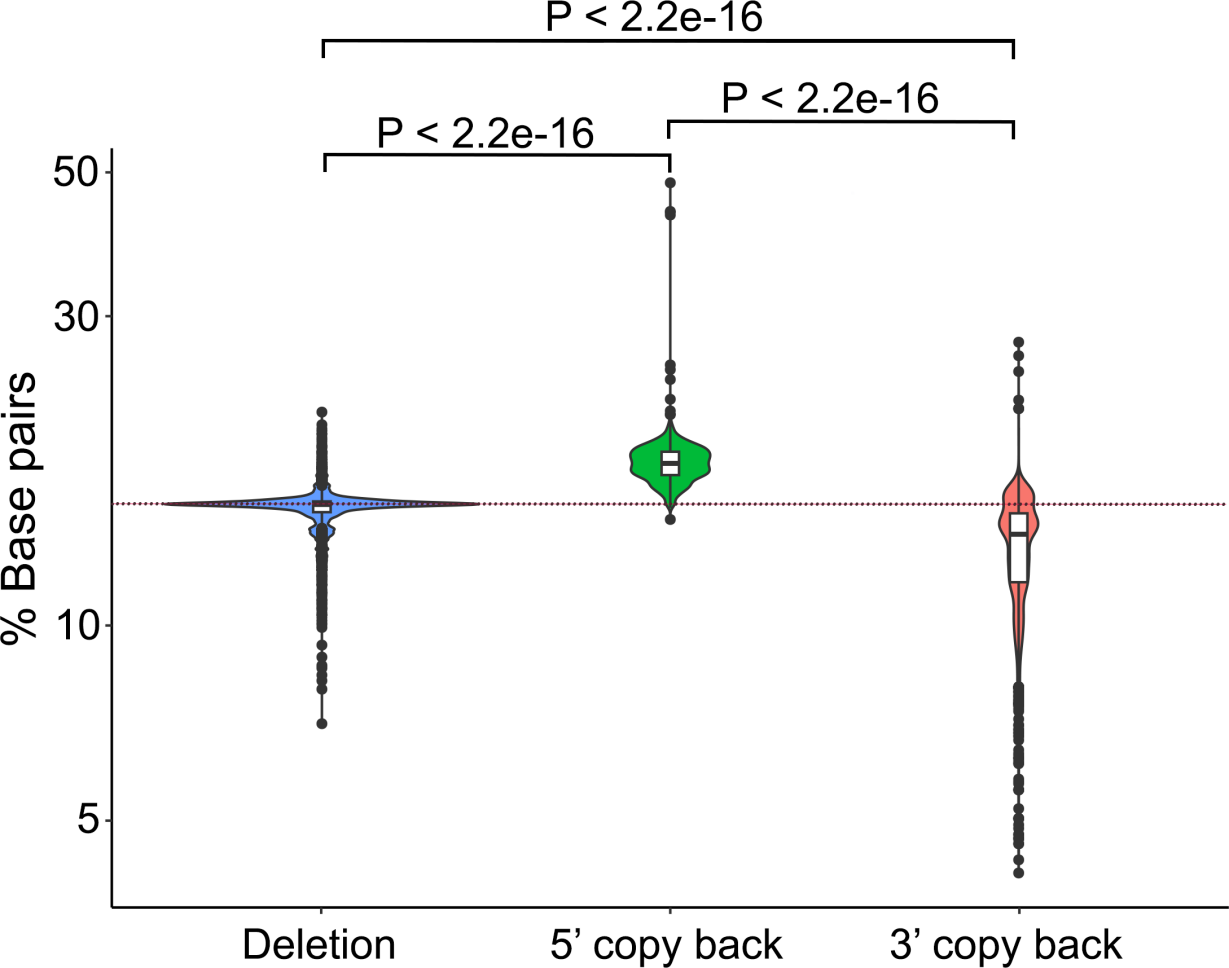
Base pair content of defective genomes (deletion, 5′ copy-back, and 3′ copy-back). The *P*-values were calculated with a one-sided Wilcoxon rank sum test as the data did not fit a normal distribution. The dashed line showed the base pair content (15.12%) of original EBOV genome.

The data indicated that the original EBOV genome is 18,956 nucleotides long, with a base pair content of 15.37%. This is similar to the predicted average base pair content in deletion defective genomes (15.12%) and higher than that in 3′ copy-back defective genomes (13.17%), but lower than that in 5′ copy-back defective genomes (17.83%) (Fig. 5). The base pair content in deletion-defective genomes is significantly lower compared to 5′ copy-back genomes and higher compared to 3′ copy-back genomes (Fig. 5). Both 5′ copy-back and 3′ copy-back defective genomes exhibited broad ranges of base pair content (Fig. 5). The majority of 3′ copy-back defective genomes have very low base pair content, diverging substantially from the median (Fig. 5).

### Defective genomes may be transmitted between individuals

**Fig 6:**
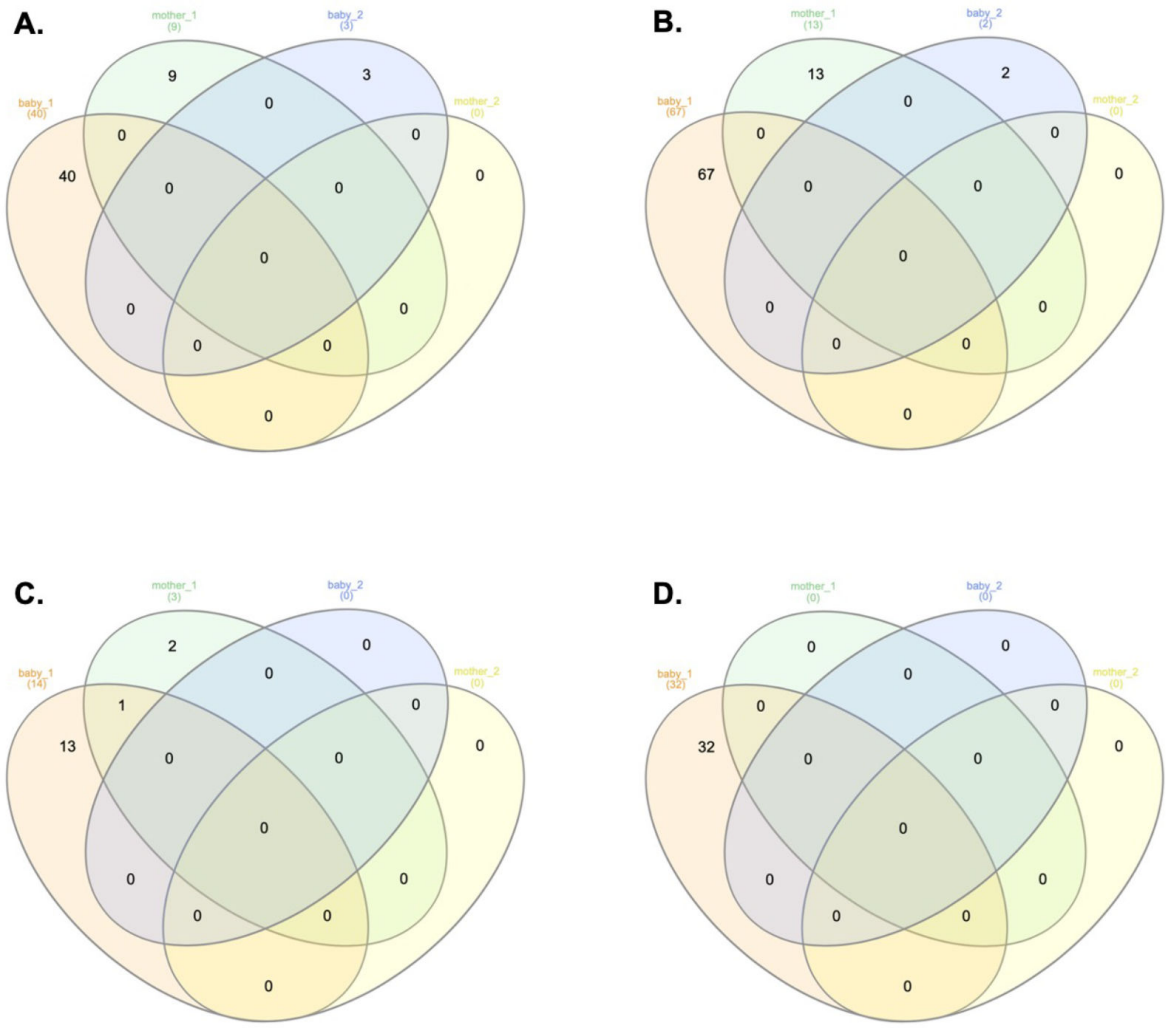
Detection of defective genomes on related patients: two mother/baby patients from EBOV outbreak 2013–2015 using DI-Tector. Diagram showing shared and unique defective genomes for both patient samples for (**A**) Deletion, (**B**) Insertion, (**C**) 5′cb, and (**D**) 3′cb defective genomes.

Defective genomes may also be transmitted between individuals. To test this hypothesis, we examined two clusters of cases that were closely related – both were mother/baby pairs, where a reasonable assumption was that the baby was infected from contact with the mother. Analysis of the viral sequencing data from these individuals indicated that sequence features of one 5′cb defective genome were shared between a mother/baby pair (Fig. 6C).

## DISCUSSION

In this study, defective genomes were identified in blood samples taken from human patients during the 2013–2016 West African EBOV outbreak. The blood samples were taken when the patients were acutely ill and at admission to an EBOV treatment center. Metadata associated with the patients included subsequent outcome of EVD – either death or survival. The tool used to identify the defective genomes was DI-tector which has been used previously to identify defective genomes in EBOV-infected NHPs (5). The bioinformatic algorithm detects sequence features that are characteristic of four different types of defective genomes: deletion, insertion, 5′cb and 3′cb defective genomes. One limitation to this approach is that the whole defective genome is not characterized but inferred by the sequence motif. However, this tool is particularly useful in examining data from short read RNA sequencing of clinical samples in outbreak settings, where the RNA was not gathered for the specific purpose of in-depth analysis and was generally fragmented and of low quality (2, 7).

Examining the sequencing data from the human blood samples with DI-tector identified the four different types of defective genomes, with the most common being insertion defective genomes in both patients who went on to survive or die from EVD. In an NHP model of EVD, the 5′ copy back type was the most abundant defective genome identified in serum taken from EBOV infected animals (5). We did not have longitudinal samples from patients and, thus, were not able to follow the abundance of a particular defective genome with time. Data collected alongside our samples were also limited due to their diagnostic nature and the resource-limited outbreak setting. In an NHP study, the same defective genomes could be detected longitudinally (5), and in our analysis of a mother/baby pair, a sequencing signature indicative of the same potential defective genome was also identified. This suggested either *de novo* synthesis of the same defective genome or perhaps more likely transmission of the same defective genome from mother to baby. We note that in our published analysis of longitudinal blood samples from a patient infected with EBOV, data suggested that the same defective genomes could be propagated with time (6). In both the NHP study (5) and that of an EVD survivor (6), defective genomes could be identified in sites described as “immune privileged,” in this case the testes and cerebral spinal fluid, respectively.

Previously, we identified that individuals who went on to die from EVD had a higher upregulation of interferon signaling compared to those who went on to survive (13). Given that in parainfluenza virus infection copyback defective genomes were shown to rapidly induce the interferon cascade (4), and in SARS-CoV-2 infection accumulation of defective genomes was seen to induce a Type I IFN response (24), we postulated that the abundance of defective genomes may have stimulated a more profound inflammatory response in patients who subsequently died of EVD rather than survived. This is likely impacted by the secondary structures formed by these defective genomes, as structural motifs are known to be readily recognized by PRRs and inform the antiviral response. Here, we report that different types of defective genomes exhibit variation in base pair content, thus likely playing a part in directing the immune response to infection (Fig. 5).

Genomic and transcription products from negative-sense RNA viruses can be detected by RIG-I. This molecule recognizes short double-stranded (i.e., base paired) RNA motifs such as panhandle structures and 5′-triphosphate ends (26). In the case of other members of the Mononegavirales order, such as measles virus or Sendai virus, defective genomes have been identified as critical PAMPs (27, 28). Our study found the base pair content of original EBOV genome is similar to the average base pair content in deletion defective genomes, which could suggest they play an important role in triggering inflammation due to their high abundance in hospitalized fatal cases compared to hospitalized survivors (Fig. 5). Therefore, these data suggest that both the abundance and profile of defective genomes may be related to the strength of the host immune response and patient outcome. Taken together, our data suggest caution should be used on potential anti-viral therapeutics based on the use of defective genomes to reduce viral load. In the case of EBOV, they may stimulate inflammatory pathways resulting in more severe disease.

## MATERIALS AND METHODS

### Sample collection, sequencing, and data collection

Sequencing data used in this project were obtained from individual blood samples taken from patients infected with EBOV by the EMLab as part of the global response to the Ebola crisis in West Africa between 2013 and 2016. Blood was drawn from patients upon admission to the Ebola Treatment Center for diagnostic purposes. For sequencing of the samples, RNA-seq libraries were prepared from the DNAse-treated total RNA using the Epicentre ScriptSeq v2 RNA-Seq Library Preparation Kit, followed by 10–15 cycles of amplification and purification using AMPure XP beads. Each library was quantified using Qubit and the size distribution assessed using the Agilent 2100 Bioanalyser, and the final libraries were pooled in equimolar ratios. The raw fastq files generated by HiSeq2500 were trimmed to remove Illumina adapter sequences using Cutadapt v1.2.1 (29). The option “−O 3” was set so that the 3′ end of any reads which matched the adapter sequence with greater than 3 bp was trimmed off. The reads were further trimmed to remove low-quality bases, using Sickle v1.200 (30) with a minimum window quality score of 20. After trimming, reads shorter than 10 bp were removed. In total, 329 samples were sequenced from this outbreak. Samples were selected for this study if the final assembled dominant genome sequence was longer than 18,800 nucleotides and contained no gaps. In total, 148 samples meeting these criteria, including 56 survivors and 92 fatal cases. The sample ID for the data used in this analysis, outcome for the patient, and EBOV viral load (Ct) are summarized in Table S1.

### Identification of defective genomes

Hisat2 v2.1.0 (31) was used to map the trimmed reads on a human reference genome assembly GRCh38 (Ensembl release-91) with default setting. The unmapped reads were extracted by bam2fastq (v1.1.0) for identification of DI events by comparing to the EBOV reference genome (GenBank sequence accession: KY426690) using DI-tector (v0.6) (12) program with default setting. The DI-tector outputs including four types of DIs (5′ cb/sb, 3′ cb/sb, Deletion, and Insertion) that were further parsed to calculate the number of different DI sites in each sample. We then applied the Spearman correlation method to compute the correlation between the rank of number of different DI sites and the rank of 1/Ct values of each sample, where −1 indicates a strong negative correlation and 1 indicates a strong positive correlation. The normality of the distribution of the data was checked by Shapiro-Wilk *W*-test (*P* > 0.05, sample size < 5000) using *shapiro.test* in the “stats” package (32).

### Prediction of RNA secondary structures in defective genomes

ScanFold (v2.0) (25) was used to analyze structures in defective genomes. ScanFold was developed to identify potential RNA structures in viral genomes and operates as a two-stage pipeline consisting of ScanFold-Scan and ScanFold-Fold (24, 25). The default settings (a 120 nt window, 1-nt step size, and mononucleotide shuffling) are recommended for most applications (25). The default window size of 120 nucleotides is optimal and has been validated as ideal for a wide range of viral genome sizes (25). Therefore, in this study, both programs were run using the default settings. The ScanFold-Fold program analyzes the output of the scanning window analysis from ScanFold-Scan, generating a list of paired bases and RNA secondary structures (filtered by average *z*-scores less than −1). The percentage of paired bases in the defective genomes was then calculated based on this filtered list.

## Data Availability

The sequencing data are available under NCBI PRJNA1251866. The NCBI SRA accession numbers are reported in Table S1.

## SUPPLEMENTAL MATERIAL

Supplemental figures jvi.00826-25-s0001.docx Figures S1 to S3.

Table S1 jvi.00826-25-s0002.xlsx Patient characteristics and NCBI accession numbers.

ASM does not own the copyrights to Supplemental Material that may be linked to, or accessed through, an article. The authors have granted ASM a non-exclusive, world-wide license to publish the Supplemental Material files. Please contact the corresponding author directly for reuse.
