# Supplementary material for: Discontinuous EBOV RNA synthesis events in patients with Ebola virus disease and their relationship to viral load and outcome of infection
Source: J Virol. 2025 Nov 11;99(12):e00826-25. doi: 10.1128/jvi.00826-25 (PMC12724386; doi:10.1128/jvi.00826-25)
Supplement: Supplemental figures — Figures S1 to S3. [file jvi.00826-25-s0001.docx]

**Supplemental Figures:**


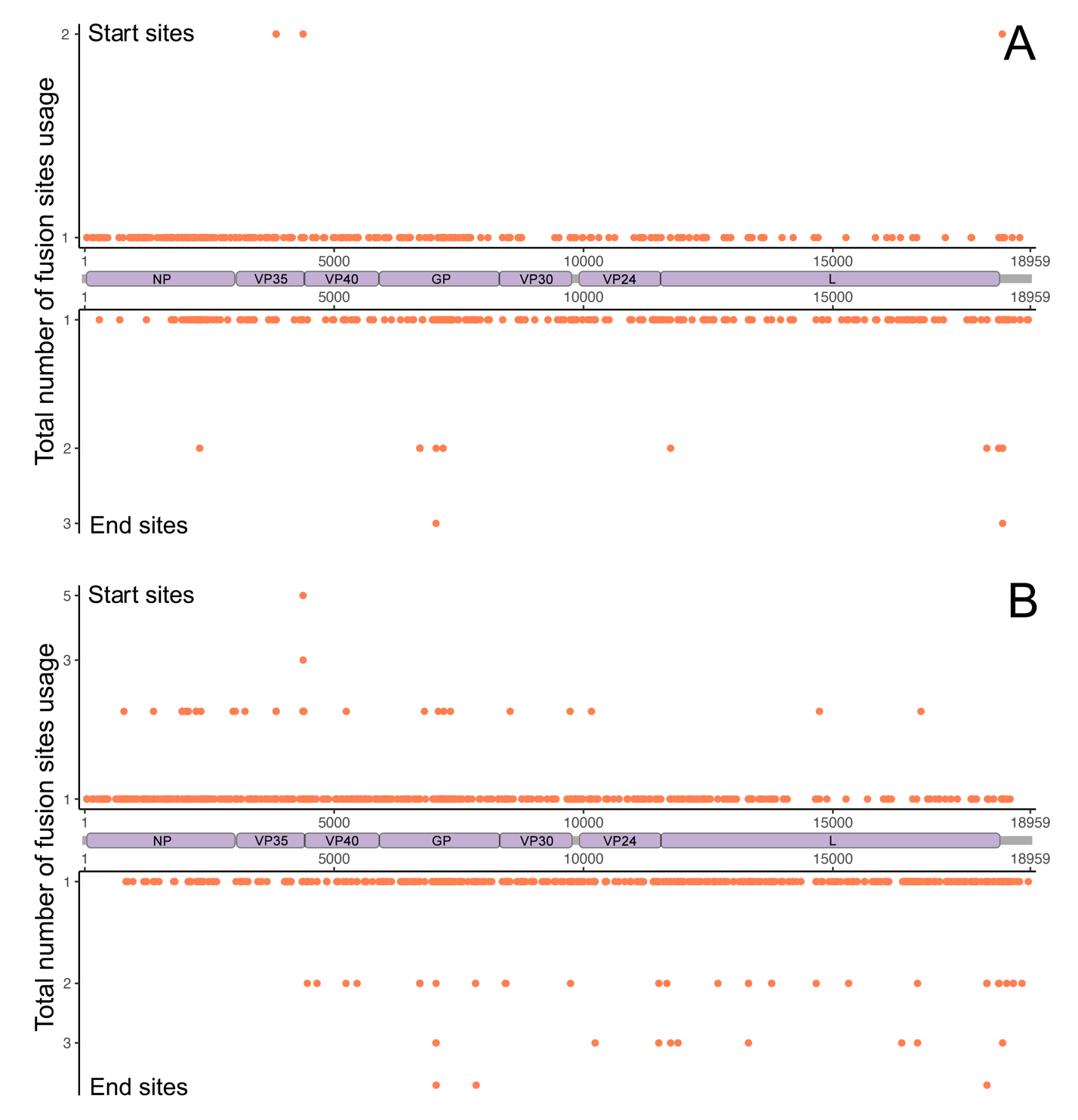


***Figure S1. Location of the fusion sites in the insertion events along the EBOV genome.*** *Scatter plot showing the total number of the fusion sites (start and end sites) in the* ***insertion*** *events along the EBOV genome identified from (A)* hospitalised survivors and *(B)* hospitalised fatal cases.


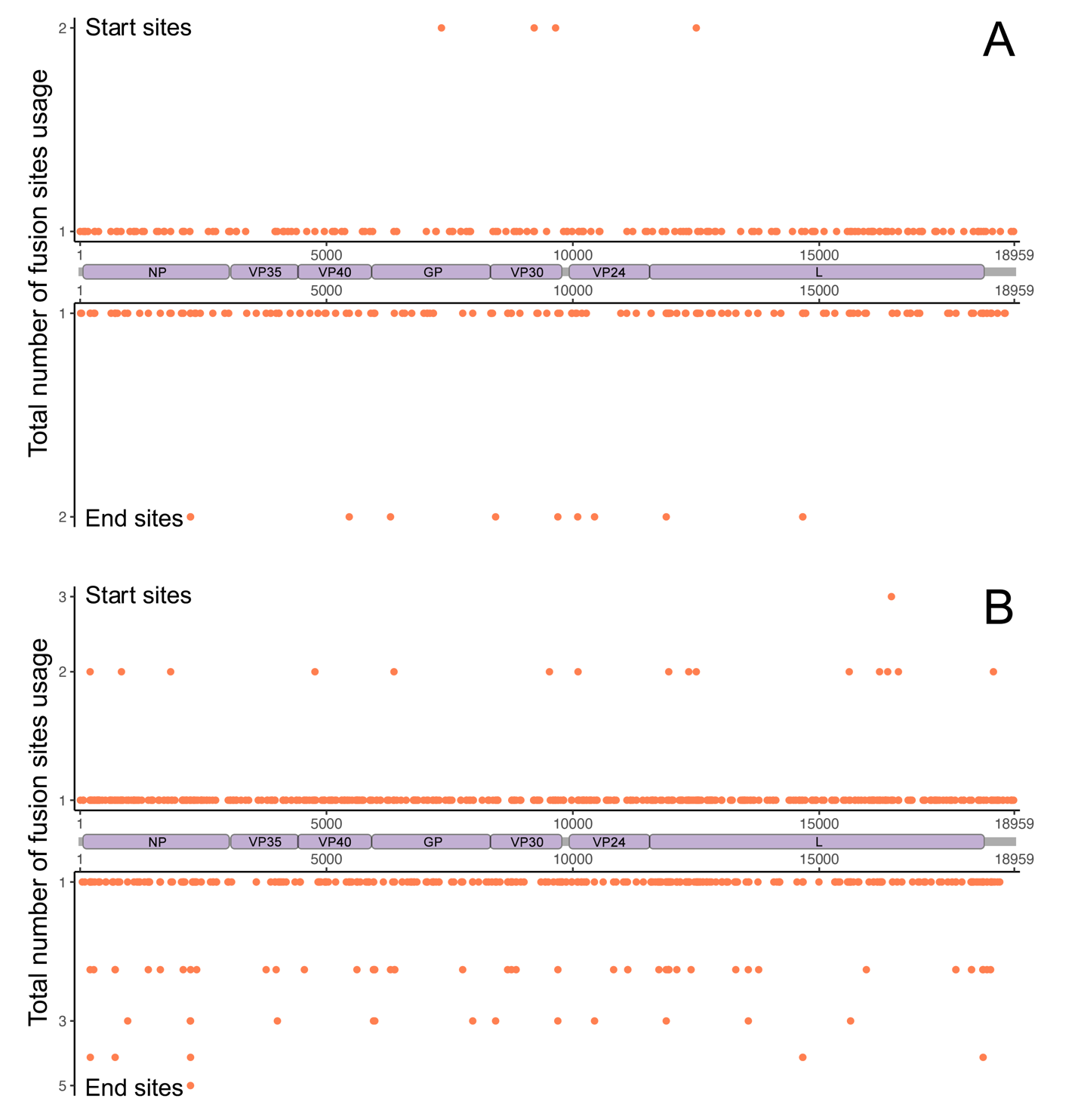


***Figure S2. Location of the*** ***fusion sites in*** *the* ***3cb events along the EBOV genome.*** *Scatter plot showing the total number of the fusion sites (start and end sites) in the* ***insertion*** *events along the EBOV genome identified from (A)* hospitalised survivors and *(B)* hospitalised fatal cases.


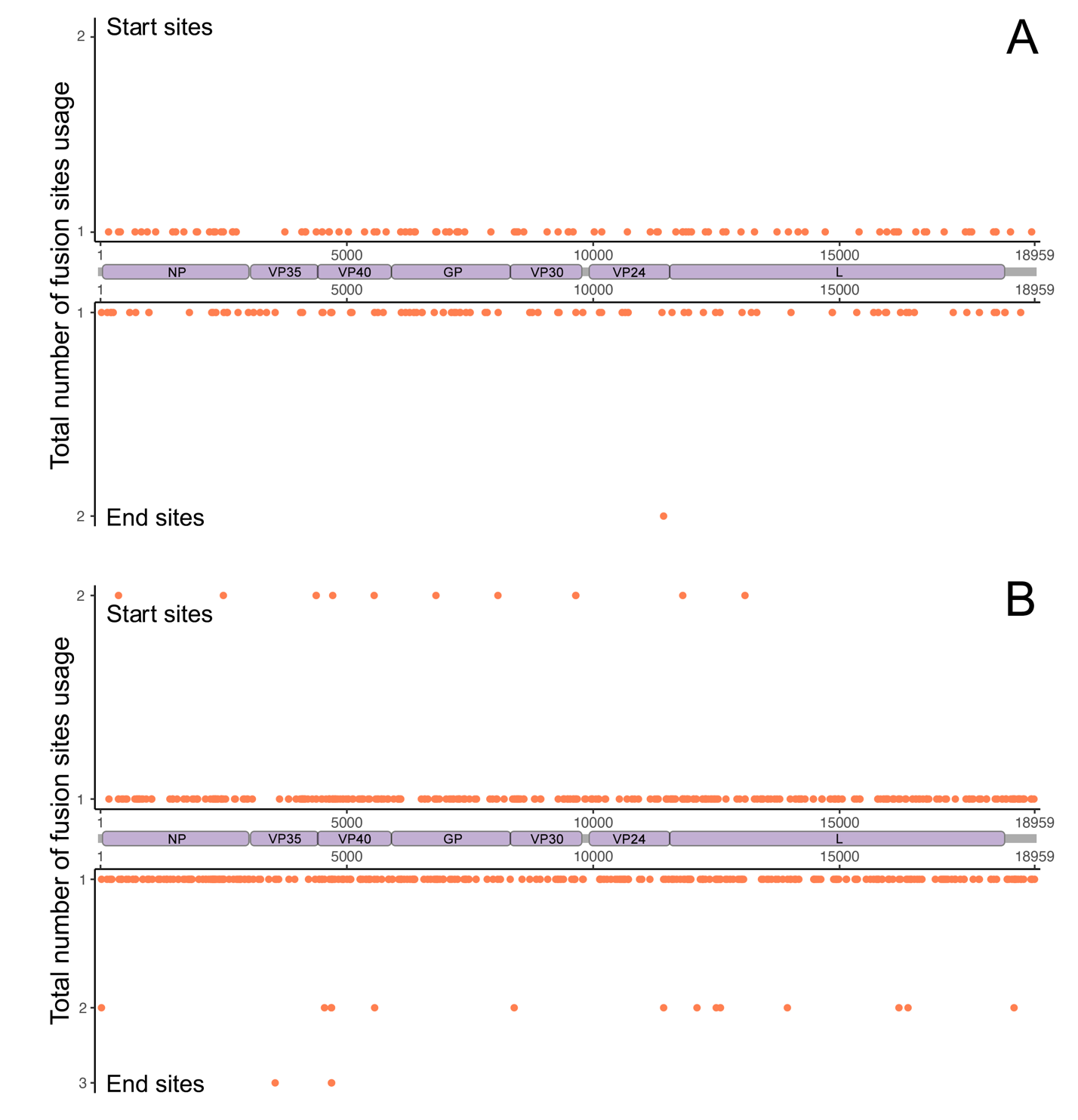


***Figure S3. Location of the fusion sites in the*** ***5cb events along the EBOV genome.*** *Scatter plot showing the total number of the fusion sites (start and end sites) in the* ***insertion*** *events along the EBOV genome identified from (A)* hospitalised survivors and *(B)* hospitalised fatal cases.
